# Preference of Traditional Bone Setting and associated factors among trauma patients with fracture at Black Lion Hospital in Addis Ababa, Ethiopia: institution based cross sectional study

**DOI:** 10.1186/s13104-019-4643-z

**Published:** 2019-09-18

**Authors:** Nardos Worku, Tsegaye Tewelde, Biru Abdissa, Hailu Merga

**Affiliations:** 1 Black Lion Hospital, Addis Ababa, Ethiopia; 2 Department of Epidemiology, Institute of Health, Jimma University, Jimma, Ethiopia; 3 Department of Nursing and Midwifery, Institute of Health, Jimma University, Jimma, Ethiopia

**Keywords:** Preference, Traditional Bone Setting, Associated factors, Black Lion Hospital, Ethiopia

## Abstract

**Objective:**

Despite the access and availability of modern health care, Traditional Bone Setting (TBS) has a big place as alternative health care. Hence, this study was aimed to assess the preference of Traditional Bone Setting and associated factors among patients with a fracture.

**Results:**

A total of 224 patients known to have fractured at Black Lion Hospital, Addis Ababa was included in the study. This study revealed that 29.9% of the study participants had a preference for the Traditional Bone Setting. Hospital admission (AOR = 8.158, 95% CI 1.179, 56.439), Traditional Bone Setting center as first port of call after injury (AOR = 0.004, 95% CI 0.001, 0.090), knowledge (AOR = 9.448, 95% CI 1.481, 60.251) and perception (AOR = 0.026, 95% CI 0.003, 0.215) were statistically significant. The preference for the Traditional Bone Setting is high. Hospital admission, Traditional Bone Setting center as a first port of call after injury, knowledge, and perception were significantly associated with the preference of Traditional Bone Setting. In addition to deployment of trained in trauma professionals, working more on awareness creation and training are recommended.

## Introduction

Fracture can occur because of trauma like a road traffic accident or a fall [1, 2]. Traditional medicine is a health practice and traditional knowledge and skill of medical aspects that passed over a generation before the era of modern medicine [3]. TBS, widely practiced all over the world before modern medicine comes into the picture, is also a known procedure among Africans and it involves the use of splints and bamboo stick or rattan cane or palm leaf axis with cotton thread or old cloth [4]. Traditional bone setter is a lay practitioner who practices management of dislocations and fractures without having had any formal training. Despite the access and availability of modern health care, Traditional Bone Setting (TBS) has a big place as an alternative health care [5]. Evidences indicated that 80% of the people in SSA use traditional medicine as a first port [6, 7]. In several studies, the reason for the preference of TBS includes easy accessibility, cultural belief, quick service, cheaper fee, pressure from friends and families and utilization of incantation and concoctions [8–13].

Evidences from Ethiopia showed half of the amputations were performed due to gangrene applied by TBS [14]. There are increasing complications like gangrene associated with TBS as a result of tightly wrapped bamboo splint application [15]. The number of trauma patients with a fracture is dramatically increasing in Ethiopia due to majorly sharp rise in the incidence of road traffic accidents. A study from Black Lion Hospital showed 58% of amputations performed for gangrene were caused by TBS tight bamboo splint [14]. Another study conducted in Addis Ababa showed the TBS as the leading cause of delay for modern treatment [16]. However, there was no finding on the efficacy of TBS in comparison to modern medicine. Therefore, this study aimed to assess the preference of TBS and associated factors among patients with a fracture which can be an eye opener to integrate them into the primary health care system.

## Main text

### Methods

#### Study design and sampling

Hospital-based cross-sectional study design was conducted in Black Lion Hospital, Addis Ababa from March 5 to April 30, 2018. It is the largest government hospital serving about 370,000–400, 000 populations a year. The Adult Orthopedic outpatient department sees around 10,000 patients a year out of which 5000 were patients with fractures. Eighteen years and above trauma patients with fracture and visiting the outpatient department during the data collection period were included in the study. However, Patients who seek medical care elsewhere before coming to the Hospital and patients with fractures other than upper extremity, lower extremity or pelvis were excluded from the study.

The sample size was determined using a single population proportion formula by considering: 95% confidence interval, 84% proportion of patients Preferring TBS as first-line treatment [17] and 5% marginal error. Then, after considering a 10% for non-response rate, 227 sample sizes was taken using systematic random sampling. On average 350 to 450 fractured patients were seen every month. Therefore, on average 700 patients were expected to visit the OPD during the study period (7 weeks) and the sample size was 227 which is one-third of the total patients expected to be seen during the study period. By taking the final sample size, patients were recruited in the study every three intervals and the first patient was selected by lottery method.

Data were collected by two Nurses using a pretested structured questionnaire developed after reviewing literature [14, 15, 17]. Face-to-face exit interview was undertaken at the Orthopedic Department Adult OPD clinic. In this study, knowledge was measured using eleven knowledge related questions and answering nine questions and above was used as a cutoff point to label them knowledgeable [18].

#### Analysis

STROBE checklist was used to analyze and report data [19]. Data were entered into EpiInfo and exported to SPSS software for analysis. Descriptive analyses were performed and binary logistic regression was performed. *p* value < 0.05 and 95% confidence interval (CI) and AOR was used in judging the statistical significance of the associations between independent variables and the outcome variable.

### Results

#### Socio-demographic characteristics

Among 227 study participants, 224 responded making the response rate 98.7%. More than half of the study participants were male and 40.2% of them were more than 40 years. One hundred seven (47.8%) of the study participants were Orthodox Christians. Majority of respondents were urban residents and more than half of them were married. More than one-third, 38.8%, of the study participants had completed secondary school (Table 1).

**Table 1:** Socio-demographic characteristics of study participants in Black Lion Hospital, 2018 (n = 224)

Variables	Frequency	Percent (%)
Sex		
Male	131	58.5
Female	93	41.5
Age in years		
18–30	81	36.2
31–40	53	23.6
> 40	90	40.2
Residency		
Urban	135	60.3
Rural	89	39.7
Marital status		
Single	62	27.7
Married	133	59.4
Separated/divorced	20	8.9
Widowed	9	4.0
Educational status		
No formal education	46	20.5
Primary school	47	21.0
Secondary school	87	38.8
Tertiary school and above	44	19.6
Occupation		
Civil servant	55	24.6
Private employee	29	12.9
Own business	47	21.0
Farmer	25	11.2
Daily laborer	15	6.7
Unemployed	53	23.7
Religion		
Orthodox	107	47.8
Protestant	52	23.2
Muslim	47	21.0
Catholic	18	8.0
Ethnicity		
Oromo	68	30.4
Amhara	74	33.0
Gurage	53	23.7
Tigre	22	9.8
Others^a^	7	3.1
Average monthly income		
≤ 4500 ETB	113	50.4
> 4500 ETB	111	49.6

^a^Harari, Somali, and Gambella ethnic groups

#### Injury and preference related factors

The study revealed that more than half of the respondents were injured due to the road traffic accident. One hundred nine (48.7%) of them were admitted to hospital for injury and from those 30.3% of them had a complication during admission (Table 2). One hundred seventy-seven (79.0%) of the study participants had no associated injury other than extremity injury. Nearly one-third, 30.3% of the study participants had a complication during admission and 27.3% of them had surgical site infection.

**Table 2:** Injury and preference related factors among study participants in Black Lion Hospital, 2018

Variables	Frequency	Percent (%)
Mechanism of injury (n = 224)		
Road traffic accident	116	51.8
Falling accident	84	37.5
Domestic accident	22	9.8
Others (gunshot, water tube fall)	2	0.9
Type of injury (n = 224)		
Upper extremity	97	43.3
Lower extremity	104	46.4
Pelvic fracture	23	10.3
Associated injury other than extremity injury (n = 224)		
Yes	47	21.0
No	177	79.0
Admission to a hospital for injury (n = 224)		
Yes	109	48.7
No	115	51.3
Complication during admission (n = 109)		
Yes	33	30.3
No	76	69.7
Condition during admission (n = 109)		
Critical	39	35.8
Stable	67	61.5
Don’t know	3	2.8
Condition during discharge (n = 109)		
Improved	86	78.7
Cured	10	9.3
Same	13	12.0
TBS center first port of call after injury (n = 224)		
Yes	114	50.9
No	110	49.1

#### Knowledge and perception

From the total, 213 (95.1%) heard about the TBS and 58.9% of the respondents knew that the drawbacks of TBS. Two hundred twenty (98.2%) of the study participants heard about modern orthopedic care; however, only 42.4% of the respondents were knowledgeable about TBS. About one-fourth, 25.9%, of the study participants perceived that TBS is better than health facilities treatment. Out of 58% who knew drawback of TBS, 36.9% of them identified lack of proper knowledge and skill as a drawback of TBS.

Most of the study participants (96.4%) thought that TBS is cheaper and 92.9% of them thought that TBS has quick services than health facilities. More than half of the respondents, (53.6%) did not think that the nearby health facilities have enough service providers. The study showed 40.6% of them had their family member/relative encountered injury in the past and in which more than half, 61.5%, of them were managed by traditional bone setters.

#### Preference of injury management and associated Factors

This study showed 29.9% (95% CI 23.7, 36.6) of them had a preference for TBS for injury management. Bivariate analysis showed; sex, residency, marital status, educational status, religion, ethnicity, average monthly income, type of injury, associated injury other than extremity injury, hospital admission for injury, TBS center first port of call after injury, knowledge, perception, fear of operation/amputation in health facility, lack of satisfaction from health facility service, time taken to reach TBS, time taken to reach modern health facility, and family/relative history of injury were significantly associated with preference of TBS among trauma patients with fracture.

Multivariable logistic regression analysis revealed those trauma patients who were not admitted in a hospital for injury were about eight times more likely to prefer TBS for injury management than those trauma patients who were admitted in a hospital for injury (AOR = 8.158, 95% CI 1.179, 56.439).

Those trauma patients who didn’t use TBS center as their first port of call after injury were 99.6% less likely to prefer TBS for injury management as compared to trauma patients who used TBS center as their first port of call after injury (AOR = 0.004, 95% CI 0.001, 0.090). Trauma patients who weren’t knowledgeable about TBS were about 9.5 times more likely to prefer TBS for injury management than those trauma patients who were knowledgeable about TBS (AOR = 9.448, 95% CI 1.481, 60.251). Trauma patients who did not perceive that TBS is better than health facility service in cure were 97.4% less likely to prefer TBS for injury management as compared to those who perceived that TBS is better than health facility service in cure (AOR = 0.026, 95% CI 0.003, 0.215) (Table 3).

**Table 3:** Factors associated with the preference of injury management among trauma patients with a fracture in Black Lion Hospital, 2018 (n = 224)

Variables	Preference		COR (95% CI)	AOR (95% CI)	p-value
	TBS	MHF			
Admission to a hospital for injury					
Yes	14	95	1.00	1.00	
No	53	62	5.801 (2.967, 11.339)	8.158 (1.179, 56.44)	0.033*
TBS center first port of call after injury					
Yes	61	53	1.00	1.00	0.001*
No	6	104	0.050 (0.020, 0.123)	0.004 (0.001, 0.090)	
Knowledge					
Knowledgeable	16	113	1.00	1.00	0.018*
Not knowledgeable	51	44	8.186 (4.227, 15.852)	9.448 (1.481, 60.25)	
TBS is better than health facility service (Perception)					
Yes	46	12	1.00	1.00	0.001*
No	13	141	0.024 (0.010, 0.056)	0.026 (0.003, 0.215)	0.184
Don’t know	8	4	0.522 (0.134, 2.029)	0.069 (0.001, 3.567)	

*TBS* Traditional Bone Setting, *MHF* modern health facility, *COR* Crude odds ratio, *AOR* adjusted odds ratio, *CI* confidence interval

* Statistically significant factors in multivariable analysis

### Discussion

This study revealed that 29.9% of the respondents preferred TBS than modern health facilities. The finding indicates that the preference of TBS is high among trauma patients with a fracture. However, this finding is lower compared to a study conducted in Kenya which indicated that 84% of respondents preferred TBS as their first choice for fracture treatment [17]. Also, it is lower than another study done in Ilorin, North Central Nigeria and Uyo, Nigeria which showed that 69.3% and 40% of study participants preferred TBS than modern medicine respectively [20, 21]. The observed difference might be the majority of the accidents in Ethiopia are road traffic related, and lead to major injuries that need urgent hospital management. In addition, road traffic-related injuries are usually covered by insurance companies and have medico-legal implications pushing for hospital-based management. However, the findings of this study was higher than the finding of the study conducted in Ghana which revealed 25% of the study participants prefer TBS than modern health facilities [18]. The possible reasons for these differences might be due to differences in the study setting, culture, and socio-demographic characteristics of study participants.

The study showed those trauma patients who were not admitted in the hospital for injury were about eight times more likely to prefer TBS for injury management than those trauma patients who were admitted in the hospital for injury management. This can be explained by the fact that those patients admitted to a hospital for injury management tend to have a major skeletal or other system injury not amenable for traditional bone setters and the care for the admitted patients at the hospital may be good enough to convince the patients to prefer and use modern health facilities. In addition, it might be due to the fact that admitted patients may have enough information about the modern management of fracture through health education or counseling in health facilities.

This study revealed that those patients who did not use TBS center as their first port of call after injury were less likely to prefer TBS for injury management as compared to trauma patients who used TBS center as their first port of call after injury. This might be due to the fact that trauma patients who used modern health care as the first portal of call are more likely to end up being managed in the same place again and again because they may develop confidence and well understand modern fracture management based on the good result of their initial visit.

As well, the study evidenced that trauma patients who weren’t knowledgeable about TBS were 9.4 times more likely to prefer TBS than those trauma patients who were knowledgeable. This might be due to knowing the real pros and cons of being managed by traditional bone setters almost always lead to a preference of modern orthopedic and trauma care.

This study also showed that trauma patients who did not perceive that TBS is better than health facility services in cure were 97.4% less likely to prefer TBS for injury management as compared to those who perceived that TBS is better than health facilities. This might be due to the fact that perception determines what individuals do, and patients who do not perceive a treatment is curable, will not seek it.

### Conclusion

This study showed that the preference of TBS is high among trauma patients with a fracture. Hospital admission for injury, TBS center as the first port of call after injury, knowledge, and perception were significantly associated with the preference of TBS. Therefore, working more on awareness creation on the need for facility-based management of trauma patients and the integration of TBS into the health care delivery system in accordance with the WHO recommendation is recommended. Moreover, training and deployment of professionals trained in trauma care to the nearest possible to improve access and quality of the service should be considered.

## Limitations

Even though this study has strengths like using standard and validated tools for data collection, it has limitations as it is the experience of a single public ortho-trauma facility (Black Lion Hospital) and the nature of cross sectional study that may underestimate cause and effect relationship.

## Data Availability

The datasets used and/or analyzed during the current study are available from the corresponding author on reasonable request.

## Abbreviations

FMOH: Federal Ministry of Health
OR: odds ratio
TM: traditional medicine
TBS: Traditional Bone Setting
WHO: World Health Organization
